# HIV infection and ART exposure affect tumor TCR repertoire of diffuse large B cell lymphoma

**DOI:** 10.1172/jci.insight.180771

**Published:** 2024-05-23

**Authors:** Sophia M. Roush, Jenny Coelho, Alexander M. Xu, Kaushik Puranam, Marriam Mponda, Edwards Kasonkanji, Maurice Mulenga, Tamiwe Tomoka, Jonathan Galeotti, Amy Brownlee, Hormas Ghadially, Maganizo Chagomerana, Blossom Damania, Matthew Painschab, Akil Merchant, Satish Gopal, Yuri Fedoriw

**Affiliations:** 1 Department of Pathology and Laboratory Medicine, School of Medicine, University of North Carolina, Chapel Hill, North Carolina, USA.; 2 Samuel Oschin Comprehensive Cancer Institute, Cedars-Sinai Medical Center, Los Angeles, California, USA.; 3 University of North Carolina Project Malawi, Lilongwe, Malawi.; 4 University of Malawi College of Medicine, Lilongwe, Malawi.; 5 University of North Carolina Lineberger Comprehensive Cancer Center, Chapel Hill, North Carolina, USA.; 6 Department of Pathology, School of Medicine and Oral Health, Kamuzu University of Health Sciences, Lilongwe, Malawi.; 7 Department of Microbiology and Immunology and; 8 Division of Hematology, School of Medicine, University of North Carolina, Chapel Hill, North Carolina, USA.; 9 Division of Hematology and Oncology, Department of Medicine, Cedars-Sinai Medical Center, Los Angeles, California, USA.; 10 National Cancer Institute Center for Global Health, Rockville, Maryland, USA.

**Keywords:** AIDS/HIV, Hematology, Lymphomas, T cell receptor

## Abstract

The most common subtype of lymphoma globally, diffuse large B cell lymphoma (DLBCL), is a leading cause of cancer death in people with HIV. The restructuring of the T cell compartment because of HIV infection and antiretroviral therapy (ART) may have implications for modern treatment selection, but current understanding of these dynamic interactions is limited. Here, we investigated the T cell response to DLBCL by sequencing the T cell receptor (TCR) repertoire in a cohort of HIV-negative (HIV^–^), HIV^+^/ART-experienced, and HIV^+^/ART-naive patients with DLBCL. HIV^+^/ART-naive tumor TCR repertoires were more clonal and more distinct from each other than HIV^–^ and HIV^+^/ART-experienced ones. Further, increased overlap between tumor and blood TCR repertoires was associated with improved survival and HIV/ART status. Our study describes TCR repertoire characteristics for the first time to our knowledge in an African DLBCL cohort and demonstrates contributions of HIV infection and ART exposure to the DLBCL TCR repertoire.

## Abstract 2

A unique cohort of patients from Malawi with diffuse large B cell lymphoma, including those who are HIV^+^/ART-naive, HIV^+^/ART-experienced, and HIV-negative revel associations between systemic immunity and T cell receptor repertoire characteristics.

## Graphical abstract

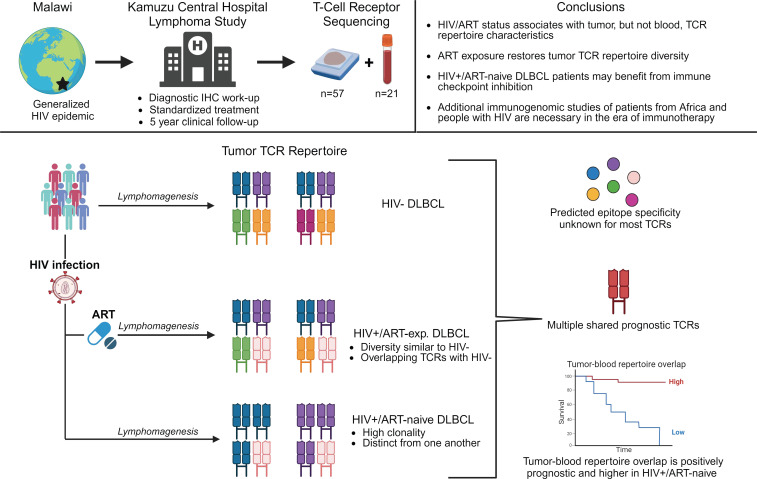

## Introduction

HIV infection alters the immune environment through oncogenic viral reactivation, decreased immune surveillance, and persistent immune activation, together contributing to increased risk of cancer, particularly aggressive B cell lymphoma (1). Diffuse large B cell lymphoma (DLBCL) is the most frequent lymphoma subtype in people with HIV and without HIV (2, 3). Effective antiretroviral therapy (ART) implementation worldwide has led to a growing and aging HIV^+^ population, such that DLBCL is now a leading cause of cancer death for people with HIV (4–6). Extensive molecular characterization has identified biologically and therapeutically meaningful subtypes of DLBCL, but these studies have excluded DLBCL arising in people with HIV (HIV^+^ DLBCL) (7–10). The distinct molecular profile of HIV^+^ DLBCL provides evidence for an impact of HIV on lymphomagenesis and immune response, and elucidating this impact is crucial for improving therapeutic paradigms for HIV^+^ patients with DLBCL (11, 12).

In the era of immunotherapy, there is interest in understanding tumor-host interactions, particularly in T cells, to improve treatment selection and outcomes for patients with cancer. Naive T cells express a unique T cell receptor (TCR) that results from gene rearrangement in the thymus (13). Upon binding to a peptide antigen presented by major histocompatibility complex (MHC) molecules of a nucleated cell (MHC class I, recognized by CD8^+^ T cells) or an antigen-presenting cell (MHC class II, recognized by CD4^+^ T cells), the T cell proliferates to mount an immune response, termed “clonal expansion” (14). TCR repertoire sequencing and the various methods to estimate bulk clonality metrics and predict antigen specificity have been thoroughly reviewed by Frank et al. (13). In brief, high TCR repertoire clonality implies much of a repertoire is composed of relatively few distinct clones that have expanded, while high TCR repertoire diversity results from many different clones in the repertoire. High clonality in the tumor TCR repertoire may indicate effective antitumor T cell response. Meanwhile, in the blood TCR repertoire, high clonality may indicate immune dysfunction (e.g., loss of CD4^+^ T cells because of untreated HIV) or response to an active infection leading to rapid expansion of select clones.

In HIV^–^ DLBCL, the presence of tumor-infiltrating lymphocytes, likely a consequence of effective tumor targeting and clonal expansion, is associated with improved prognosis (15). TCR repertoires have been investigated to decipher intra- and intertumor immune heterogeneity, which may have implications for biomarker development (13). Tumor and whole-blood TCR repertoire diversity, as well as the degree of overlap between these compartments, have been assessed as predictive and prognostic biomarkers in a variety of solid tumors (16). In a cohort of HIV^–^ patients with DLBCL treated with conventional chemotherapy, higher intratumor TCR repertoire diversity was positively prognostic (17).

In the setting of HIV infection, systemic immune response is dysregulated through a decrease in the CD4^+^ T cell compartment and chronic stimulation and exhaustion of CD8^+^ T cells (18). Over time, HIV infection results in decreased TCR repertoire diversity, in part due to an expansion of clones targeting common HIV epitopes during chronic infection (19). Though ART restores T cell function and immune response, patients have varying responses (20–22). Furthermore, the impact of HIV infection and ART exposure on the DLBCL TCR repertoire has not been investigated.

Forming generalizable conclusions regarding antitumor immune responses in HIV^+^ DLBCL in high-income countries is difficult because of a disproportionate HIV burden on men who have sex with men and racial/ethnic minorities (23). Our cohort offers distinctive opportunities to investigate DLBCL biology by HIV and ART status given the generalized HIV epidemic and effective ART scale-up effort in Malawi, as well as the substantial number of HIV^+^ and HIV^–^ lymphomas at our site (6). Importantly, our cohort has relatively few EBV^+^ DLBCLs, allowing us to focus on effects of HIV, rather than EBV, on immune response to tumor (24). In this study, enabled by longstanding clinical research collaboration focused on cancer in a setting with extremely limited public sector health care resources and high HIV prevalence, we aimed to characterize the TCR repertoire of DLBCL under varying degrees of immune pressure.

## Results

### Patient characteristics.

Our TCR-sequencing cohort consisted of 62 patients, *n* = 16 (26%) of which had paired tumor and whole blood for analysis (Table 1). The median age was 46 years and the median overall survival (OS) was 14 months. HIV^+^/ART-naive patients had a median OS of 50 months, compared with 10 months for HIV^+^/ART-exp. patients and 23 months for HIV^–^ patients. For patients who were HIV^+^, the median time on ART before DLBCL diagnosis was 36 months (0.2 months for the ART-naive cohort and 58 months for the ART-exp. cohort). The median HIV viral load was 0 copies in HIV^+^/ART-exp. patients compared with 9,800 copies in HIV^+^/ART-naive. Among patients, 32% received rituximab with an even distribution across HIV/ART status. All patients had corresponding transcriptomics sequencing data, except for 1 HIV^–^ patient. A total of 30 (48%) tumors were GC type by GEP. The HIV^–^ tumor without gene expression profiling data was predicted to be non-GC type by the Hans algorithm (25). Only *n* = 6 (10%) tumors were EBV^+^ by EBER in situ hybridization (ISH). A total of 57 pretreatment DLBCL tumors (*n* = 19 HIV^–^, *n* = 27 HIV^+^/ART-exp., *n* = 11 HIV^+^/ART-naive) and 21 pretreatment whole-blood samples (*n* = 7 HIV^–^, *n* = 7 HIV^+^/ART-naive, *n* = 7 HIV^+^/ART-exp.) from the 62 patients in the cohort were included for TCR sequencing. All patients were followed for up to 5 years or censored at the time of analysis with no loss to follow-up.

### TCR-sequencing output.

The median number of total templates was 385 for tumor and 27,000 for blood samples, with a similar percentage of productive templates in each (77% tumor, 80% blood). Further TCR metrics are in Table 2. Only productive templates were considered for the analyses in this manuscript. Because of the difference in median total productive templates (20,700 in blood vs. 300 in tumor), random downsampling to the lowest value above the 100-template threshold (*n* = 108, averaged over 100 iterations) was performed on all samples with more than 100 productive templates to account for differences in T cell and template input. Thus, further bulk clonality and diversity metric comparisons were not affected by T cell count. Thirty-five tumors (*n* = 12 HIV^–^, *n* = 8 HIV^+^/ART-naive, *n* = 15 HIV^+^/ART-exp.) and 17 whole-blood samples (*n* = 6 HIV^–^, *n* = 7 HIV^+^/ART-naive, *n* = 4 HIV^+^/ART-exp.) met inclusion criteria for clonality analysis (Supplemental Table 1; supplemental material available online with this article; https://doi.org/10.1172/jci.insight.180771DS1). Of tumors that met criteria for clonality analysis, 2 were EBV^+^ by EBER ISH (*n* = 1 HIV^+^/ART-exp., *n* = 1 HIV^–^).

### Tumor TCR repertoires have increased large TCR expansions.

**Figure 1:**
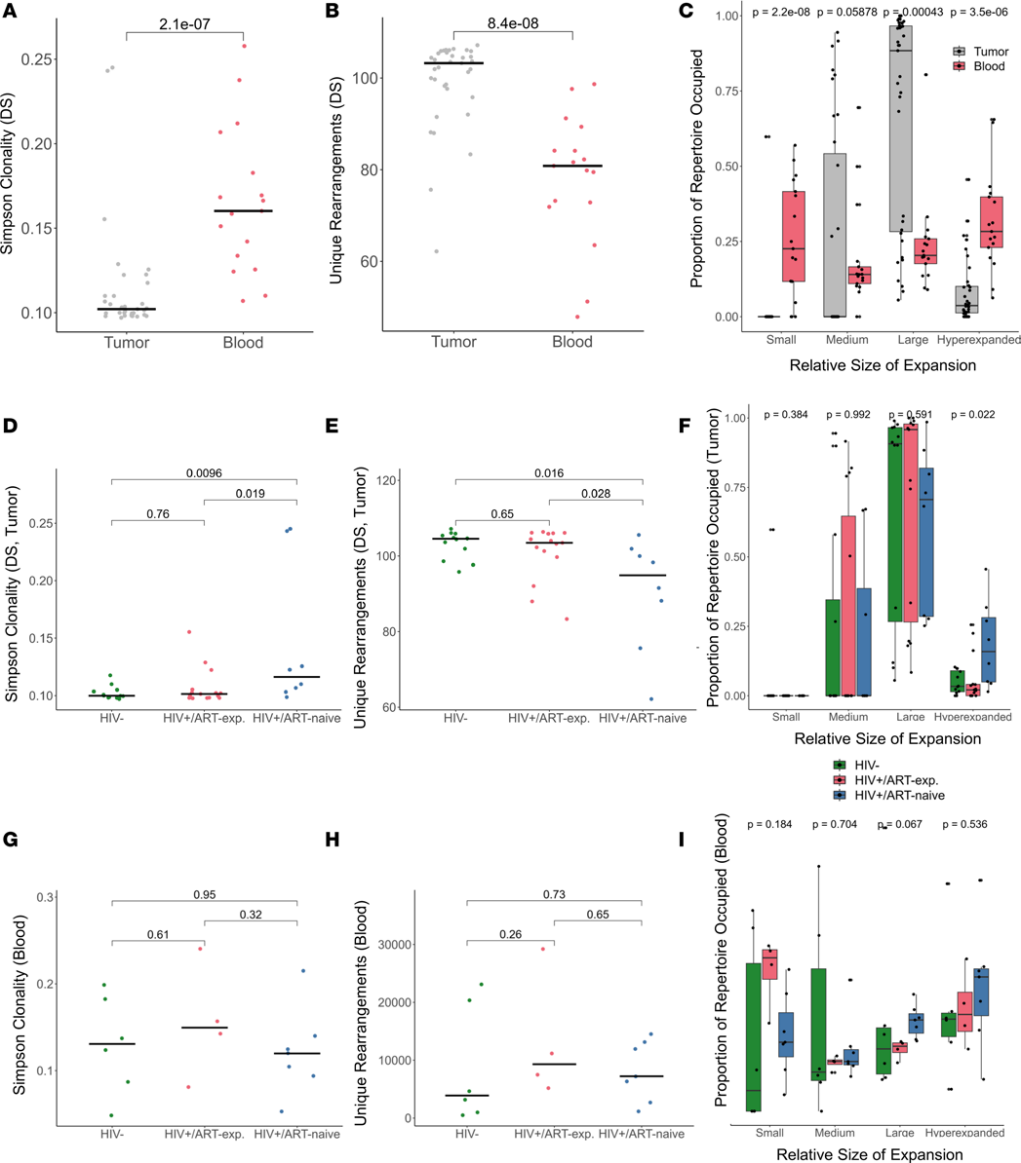
HIV/ART status is associated with tumor, but not blood, TCR repertoire bulk clonality metrics. (**A**) Simpson clonality by tissue type (*n* = 52, Wilcoxon rank sum test). Blood TCR repertoires had higher Simpson clonality compared with tumor. (**B**) Unique rearrangements by tissue type (*n* = 52, Wilcoxon rank sum test). Tumor TCR repertoires had more unique rearrangements compared with blood. (**C**) Relative abundance of TCRs with specific frequencies by sample type (*n* = 52, Wilcoxon rank sum test). Blood TCR repertoires had more hyperexpanded clones and small expansions compared with tumor, while tumor TCR repertoires had more large expansions. (**D**) Tumor Simpson clonality by HIV/ART status (*n* = 35, pairwise Wilcoxon rank sum test). HIV^+^/ART-naive tumor repertoires were more clonal compared with HIV^+^/ART-exp. and HIV^–^. (**E**) Tumor unique rearrangements by HIV/ART status (*n* = 35, pairwise Wilcoxon rank sum test). HIV^+^/ART-naive tumor repertoires had fewer unique rearrangements compared with HIV^+^/ART-exp. and HIV^–^. (**F**) Relative abundance of tumor TCRs with specific frequencies by HIV/ART status (*n* = 35, Kruskal-Wallis test). HIV^+^/ART-naive tumor TCR repertoires had more hyperexpanded clones. (**G**) Blood Simpson clonality by HIV/ART status (*n* = 17, pairwise Wilcoxon rank sum test). Similar blood TCR repertoire clonality among HIV/ART groups. (**H**) Blood unique rearrangements by HIV/ART status (*n* = 17, pairwise Wilcoxon rank sum test). Similar blood TCR repertoire diversity among HIV/ART groups. (**I**) Relative abundance of blood TCRs with specific frequencies by HIV/ART status (*n* = 17, Kruskal-Wallis test). Only productive templates were considered. Box plots show the interquartile range, median (line), and minimum and maximum (whiskers). DS indicates metrics were downsampled (**A**, **B**, **D**, and **E**). Horizontal black line indicates median (**A**, **B**, **D**, **E**, **G**, and **H**).

We first compared overall TCR clonality by tissue type. Blood TCR repertoires were more clonal (productive Simpson clonality: 1.6-fold, *P* < 0.001; max productive frequency: 3.6-fold, *P* < 0.001; Wilcoxon rank sum test) and had fewer unique productive rearrangements (0.78-fold, *P* < 0.001, Wilcoxon rank sum test) compared with tumor repertoires (Figure 1, A and B). To further investigate the bulk clonality metrics of the repertoire, we classified each clonal expansion based on the proportion of repertoire it occupied (small: 0–0.0001, medium: 0.0001–0.001, large: 0.001–0.01, or hyperexpanded: 0.01–1) without downsampling. Blood TCR repertoires had higher mean proportions of repertoire composed of small expansions (14.8-fold, *P* < 0.001, Wilcoxon rank sum test) and hyperexpanded clones (3.8-fold, *P* < 0.001, Wilcoxon rank sum test) compared with tumor. Meanwhile, tumor TCR repertoires had higher mean proportions of repertoire composed of large expansions (2.8-fold, *P* < 0.001, Wilcoxon rank sum test) than blood TCR repertoires (Figure 1C). Stratifying by size of clonal expansion revealed that blood TCR repertoires were heavily enriched for small expansions, likely representing background immune diversity, with some enrichment for hyperexpanded clones, which could be related to infection or tumor response. Meanwhile, tumor TCR repertoires were characterized by large TCR expansions, potentially indicative of effective antitumor immune response.

### HIV^+^/ART-naive DLBCL TCR repertoires are more clonal than HIV^+^/ART-exp. and HIV^–^ DLBCL.

We next tested for associations between bulk TCR repertoire metrics and the following covariates: HIV status, HIV/ART status, age, sex, HIV viral load, CD4^+^ T count, ART duration, LDH, ECOG score, stage > 2, Ki-67, EBER, and cell of origin. In tumor, only HIV/ART status was associated with bulk TCR repertoire metrics (p.adj = 0.043 for productive Simpson clonality, max productive frequency, and unique productive rearrangements; Kruskal-Wallis test with Benjamini-Hochberg [BH] correction). There was no association between HIV/ART status and total productive template count (*P* = 0.86; Kruskal-Wallis test with BH correction). In blood, there were no statistically significant associations between clonality metrics and any of the tested covariates.

As it was the only covariate that associated with bulk TCR clonality metrics, we aimed to further investigate the relationship between HIV/ART status and TCR repertoire clonality. Although total productive template count was similar by HIV/ART status, essentially normalizing for any differences in T cell presence in tumor, HIV^+^/ART-naive tumors had more clonal TCR repertoires than HIV^–^ tumors (productive Simpson clonality: 1.4-fold, *P* = 0.0096; max productive frequency: 3.1-fold, *P* = 0.012; unique productive rearrangements: 0.88-fold, *P* = 0.016; pairwise Wilcoxon rank sum test) and HIV^+^/ART-exp. (productive Simpson clonality: 1.4-fold, *P* = 0.019; max productive frequency: 2.5-fold, *P* = 0.026; unique productive rearrangements: 0.90-fold, *P* = 0.028; pairwise Wilcoxon rank sum test) (Figure 1, D and E). Interestingly, there was no difference between HIV^–^ and HIV^+^/ART-exp. tumor clonality. HIV^+^/ART-naive tumors had a higher proportion of clones that were hyperexpanded compared with HIV^+^/ART-exp. and HIV^–^ tumors (*P* = 0.022, pairwise Wilcoxon rank sum test) (Figure 1F). In blood, there were no differences in unique productive rearrangements, productive Simpson clonality, maximum productive frequency, or proportion of clonal expansion by HIV/ART status (Figure 1, G–I). Taken together, tumor TCR clonality, but not blood TCR clonality, was associated with HIV/ART status. HIV^+^/ART-naive DLBCL TCR repertoires were the most clonal, and ART may restore TCR diversity in HIV^+^/ART-exp. tumors to be similar to HIV^–^ DLBCL.

### High unique productive rearrangements in blood associate with improved survival.

**Figure 2:**
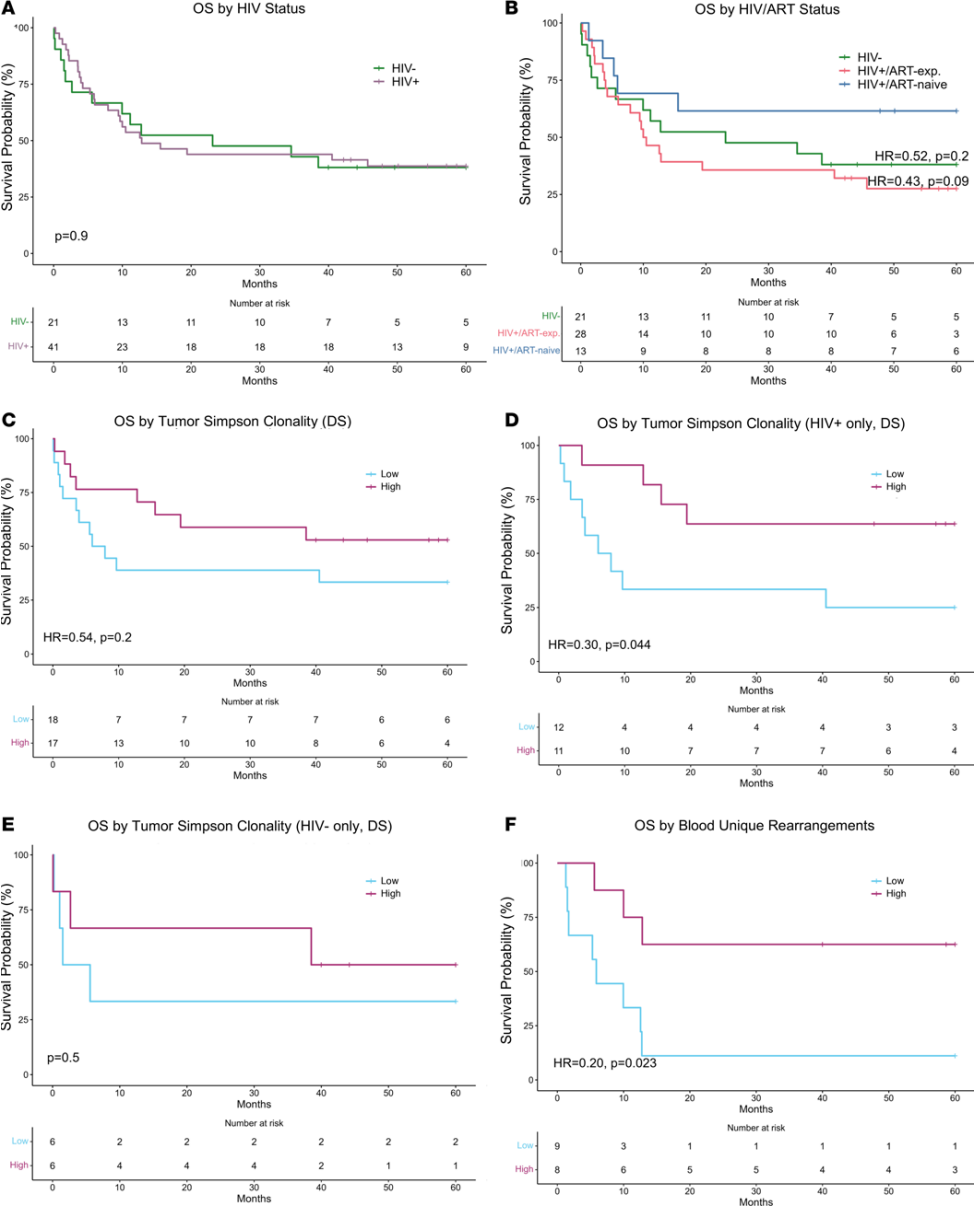
Increased tumor TCR repertoire clonality is associated with improved survival in HIV^+^ patients. (**A**) Kaplan-Meier curve of OS by HIV status (*n* = 62, Cox regression). Patients in this sequencing cohort had similar OS by HIV status. (**B**) Kaplan-Meier curve of OS by HIV/ART status (*n* = 62, Cox regression). HIV^+^/ART-naive patients trended toward improved OS compared with HIV^+^/ART-exp. in this sequencing cohort. (**C**) Kaplan-Meier curve of OS by tumor Simpson clonality (*n* = 35, Cox regression adjusted for age and HIV/ART status). Among all patients in the clonality data set, there was no statistically significant difference in OS by tumor Simpson clonality. (**D**) Kaplan-Meier curve depicting OS of HIV^+^ patients by tumor Simpson clonality (*n* = 23, Cox regression). Among HIV^+^ patients in the clonality data set, high tumor Simpson clonality was positively associated with OS. (**E**) Kaplan-Meier curve of OS of HIV^–^ patients by tumor Simpson clonality (*n* = 12, Cox regression). Among HIV^–^ patients in the clonality data set, there was no statistically significant difference in OS by tumor Simpson clonality. (**F**) Kaplan-Meier curve of OS by unique rearrangements in the blood (*n* = 17, Cox regression). Increased number of unique rearrangements in the blood was positively prognostic. High versus low as determined by median cutoff (**C**–**F**). Only productive templates were considered.

HIV status was not associated with OS (*P* = 0.9, Cox regression, Figure 2A) or progression-free survival (PFS) (*P* = 0.8, Cox regression) in the entire TCR-sequencing cohort (*n* = 62 patients). HIV/ART-naive patients trended toward improved OS and PFS compared with patients who were HIV^+^/ART-exp. (OS: HR = 0.43, *P* = 0.09; PFS: HR = 0.38, *P* = 0.054; Cox regression) or HIV^–^ (OS: HR = 0.52, *P* = 0.2, PFS: HR = 0.47, *P* = 0.14; Cox regression) (Figure 2B). Tumor TCR repertoire clonality may represent the strength and efficacy of the T cell response, and as such, affect patient outcome. When analyzing HIV^+^ and HIV^–^ tumors together, none of the clonality metrics associated with OS or PFS (Figure 2C, PFS not shown). When analyzing HIV^+^ tumors only, increased clonality was associated with improved OS (productive Simpson clonality: HR = 0.30, *P* = 0.044; max productive frequency: HR = 0.33, *P* = 0.064; Cox regression) and PFS (productive Simpson clonality: HR 0.25, *P* = 0.021; max productive frequency: HR = 0.28, *P* = 0.034; Cox regression) (Figure 2D). These were not significant after adjusting for ART status. When analyzing HIV^–^ tumors only, there was no difference in survival by clonality (*P* = 0.5, Cox regression) (Figure 2E). We also assessed for associations between blood TCR repertoire clonality metrics and survival. Having a more diverse TCR repertoire in blood correlated with improved OS (unique productive rearrangements: HR = 0.20, *P* = 0.023, Cox regression) and remained significant after adjusting for age and HIV/ART status (HR = 0.21, *P* = 0.044, Cox regression) (Figure 2F). There was no association with survival for productive Simpson clonality and maximum productive frequency. In sum, patients with high diversity of their blood TCR repertoire had improved survival, suggesting diversity of the blood TCR repertoire may act as a surrogate for overall immune health. High tumor TCR clonality may be positively prognostic among HIV^+^ patients with DLBCL in our cohort, though the association lost significance upon adjusting for ART exposure.

### The majority of TCRs in our cohort have not been previously reported.

We used VDJdb, a repository for antigen-specific TCR sequences, as a resource to explore the known antigen specificity of sequenced TCRs. On average, only 5% of tumor and 4% of blood TCRs for a given sample were found in the VDJdb database, limiting conclusions about epitope specificity of the TCRs in our cohort. The proportion of tumor TCRs in VDJdb was higher in HIV^+^/ART-naive samples compared with HIV^+^/ART-exp. (1.4-fold, *P* = 0.04, pairwise Wilcoxon rank sum test) and HIV^–^ (1.8-fold, *P* = 0.02, pairwise Wilcoxon rank sum test) (Supplemental Figure 1A). There was no statistically significant difference in HIV epitope proportion by HIV/ART status, though HIV^+^/ART-naive tumors trended toward a higher proportion of HIV epitopes compared with HIV^–^ (median 0.05 vs. 0.00, *P* = 0.09, pairwise Wilcoxon rank sum test, Supplemental Figure 1B). Influenza A, CMV, and EBV were the most common predicted epitopes (Supplemental Figure 1C). In blood, the proportion of TCRs identified in VDJdb was higher in HIV^+^/ART-exp. compared with HIV^–^ (1.6-fold, *P* = 0.04, pairwise Wilcoxon rank sum test, Supplemental Figure 1D). There was no difference in blood HIV epitope proportion by HIV/ART status (Supplemental Figure 1E), and HIV viral load did not associate with proportion of HIV epitopes in blood or tumor.

As CMV, Influenza A, and EBV were the most common predicted epitopes (Supplemental Figure 1F), we tested for differences among these species in our data set. HIV/ART-naive patients had a smaller proportion of influenza A–targeting TCRs in blood compared with HIV^+^/ART-exp. (0.54-fold, *P* = 0.007, pairwise Wilcoxon rank sum test). HIV/ART-naive blood had a smaller proportion of influenza A–targeting TCRs compared with HIV^+^/ART-exp. (0.54-fold, *P* = 0.007, pairwise Wilcoxon rank sum test) and a higher proportion of CMV-targeting TCRs compared with HIV^–^ blood (1.3-fold, *P* = 0.02, pairwise Wilcoxon rank sum test). Given the notable lack of TCRs identifiable in samples from Africa, there is a need for further TCR-sequencing studies, including non-HIV-specific ones, in the region.

Inability to identify a patient’s TCR in a database such as VDJdb may indicate lack of antigen-specific response or underrepresentation of their human leukocyte antigen (HLA) alleles, as HLA genotype affects the epitopes that can be presented by antigen-presenting cells. We performed HLA typing from transcriptome data, and the most frequent HLA-A and HLA-B alleles in our cohort (HLA-A*68, HLA-B*58) are prevalent in African populations and have been implicated in response to HIV infection (Supplemental Figure 2) (26–28). Additionally, aside from HLA-A*68 and HLA-B*07, alleles that are common in North American and northern/western European populations were not among the most frequent in our cohort (HLA-A*01, HLA-A*03, HLA-A*31, HLA-B*35, HLA-B*39, HLA-B*44, HLA-B*48) (27). As expected, the patients in our Malawi-based cohort have HLA genotypes that reflect their African ancestry, indicating underrepresentation of patients with African ancestry in common immunogenomics databases.

### HIV^+^/ART-exp. and HIV^–^ tumor TCR repertoires demonstrate overlapping TCR specificity.

After establishing associations between bulk TCR repertoire clonality and HIV/ART status, we aimed to discern differences at the TCR level, including all sequenced productive TCRs, regardless of presence in VDJdb. In order to investigate the specificity of the TCRs that compose each repertoire, rather than comparing bulk metrics, we included all sequenced samples and did not perform downsampling on the output. Thus, we cannot accurately compare bulk clonality or diversity metrics across samples or tissue types in this section.

**Figure 3:**
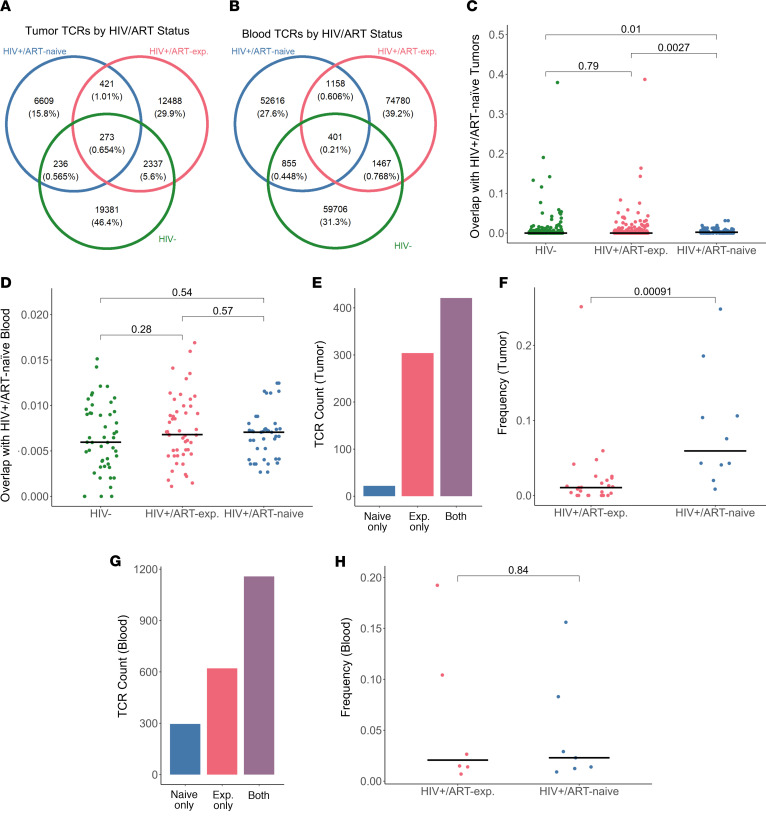
HIV^+^/ART-exp. tumor TCR repertoires are more similar to HIV^–^ than HIV^+^/ART-naive. (**A**) Distribution of unique productive tumor TCRs by HIV/ART status (*n* = 57). Most identified TCRs were found in a single HIV/ART group, with a considerable proportion of TCRs in both HIV^+^/ART-exp. and HIV^–^ tumors. (**B**) Distribution of unique productive blood TCRs by HIV/ART status (*n* = 21). Most identified TCRs were found in a single HIV/ART group. (**C**) Tumor TCR repertoire overlap by HIV/ART status with reference to HIV^+^/ART-naive tumors (*n* = 57, pairwise Wilcoxon rank sum test). HIV^+^/ART-naive tumors have greater TCR repertoire overlap with HIV^+^/ART-exp. and HIV^–^ tumors than each other. (**D**) Blood TCR repertoire overlap by HIV/ART status with reference to HIV^+^/ART-naive blood (*n* = 21, pairwise Wilcoxon rank sum test). Degree of blood TCR repertoire overlap was similar between HIV^+^/ART groups. (**E**) Number of unique TCRs found exclusively in ≥2 HIV^+^ tumor samples by ART status (*n* = 33, Wilcoxon rank sum test). There were few exclusively HIV^+^/ART-naive shared tumor TCRs. (**F**) Sample frequency of TCRs found exclusively in ≥2 HIV^+^ tumor samples by ART status (*n* = 33, Wilcoxon rank sum test). ART-naive tumors had higher frequencies of shared HIV^+^ exclusive TCRs also found in ART-exp. tumors compared with ART-exp. by Wilcoxon rank sum test. (**G**) Number of unique TCRs found exclusively in ≥2 HIV^+^ blood samples by ART status (*n* = 11). Most shared exclusively HIV^+^ blood TCRs were found in both ART-naive and ART-exp. (**H**) Sample frequency of TCRs found exclusively in ≥2 HIV^+^ blood samples (*n* = 11, Wilcoxon rank sum test). ART-naive and ART-exp. blood had similar levels of shared HIV^+^ TCRs found in both ART groups. Percentage of total unique tumor TCRs sequenced indicated in parentheses (**A** and **B**). Horizontal black line indicates median (**D**, **F**, and **H**).

In tumor, 273 unique TCRs were found in samples from all 3 HIV/ART groups, while 19,518 were specific to HIV^+^ only and 19,381 to HIV^–^ only. In blood, 401 unique TCRs were found in samples from all 3 HIV/ART groups, while 128,554 were specific to HIV^+^ only and 59,706 to HIV^–^ only. Only 17% of HIV^+^/ART-exp. tumor TCRs occurred in HIV^–^ tumors, compared with only 5% occurring in HIV^+^/ART-naive (Figure 3A). There was a 2%–3% overlap of blood TCRs across all 3 HIV/ART groups (Figure 3B). In tumor, HIV^+^/ART-exp. repertoires shared a greater proportion of TCRs with HIV^–^ compared with HIV^+^/ART-naive repertoires, providing evidence for immune reconstitution affecting tumor–T cell response in DLBCL.

### HIV^+^/ART-naive tumor TCR repertoires are distinct from one another.

Next, we examined TCR repertoire overlap by HIV/ART status at the sample level. In tumor, HIV^+^/ART-naive tumor repertoires had increased mean overlap with HIV^+^/ART-exp. (1.7-fold, *P* = 0.003, pairwise Wilcoxon rank sum test) and HIV^–^ (2.3-fold, *P* = 0.01, pairwise Wilcoxon rank sum test) compared with other HIV^+^/ART-naive repertoires (Figure 3C). In blood, there was no difference in repertoire overlap with HIV^+^/ART-naive repertoires by HIV/ART status (Figure 3D).

As expected, most of the blood and tumor TCRs in our cohort were “private,” only occurring in 1 patient. In tumor, 37,790 TCRs were found in only 1 sample, 123 were found in at least 5 samples, and 1 TCR (CAITDTGQGSYEQYF, not in VDJdb) was found in 10 samples. In blood, 185,887 TCRs were found in only 1 sample, 124 were found in at least 5 samples, and 1 TCR (CASSLGETQYF, predicted to target CMV) was found in 13 samples.

After observing low repertoire overlap among HIV^+^/ART-naive tumors, we aimed to assess whether HIV^+^/ART-naive TCRs were more likely private to 1 patient compared with HIV^+^/ART-exp tumors. We found 2,075 blood and 747 tumor shared TCRs exclusively in HIV^+^ cases. Indeed, of TCRs found exclusively in at least 2 HIV^+^ tumors, only 22 TCRs (3%) were found only in HIV^+^/ART-naive tumors compared with 350 (41%) in HIV^+^/ART-exp (Figure 3E). At the sample level, HIV^+^/ART-naive tumors had a higher proportion of shared TCRs that were also found in HIV^+^/ART-exp. (5.7-fold, *P* < 0.001, Wilcoxon rank sum test) (Figure 3F). Meanwhile, in the blood, there was no difference in frequency of TCRs found exclusively in at least 2 HIV^+^ blood repertoires by ART status (Figure 3, G and H). Taken together, the tumor, but not blood, TCR repertoires of HIV^+^/ART-naive samples were distinct from one another.

### Multiple shared tumor and blood TCRs associate with outcome.

**Figure 4:**
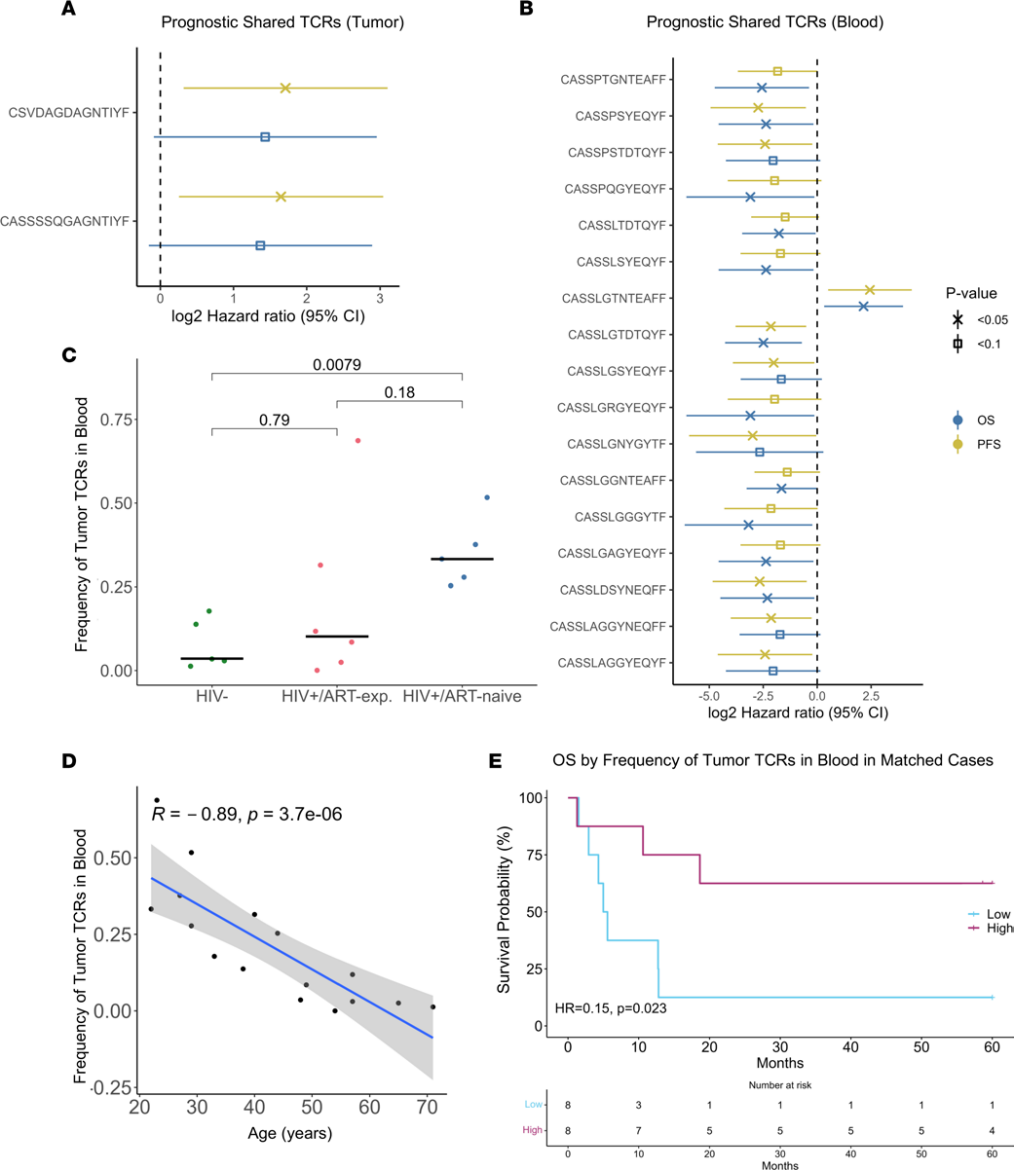
Overlap of tumor and blood TCR repertoires associates with HIV/ART status and survival. (**A**) TCRs shared by at least 5 tumor samples that associated with overall (blue) and/or progression-free (yellow) survival by univariate Cox regression. (**B**) TCRs shared by at least 5 blood samples that associated with overall (blue) and/or progression-free (yellow) survival by univariate Cox regression. (**C**) Frequency of tumor TCRs found in blood for patients with matched tumor and blood samples by age (*n* = 16, Spearman correlation). Age negatively associated with frequency of tumor TCRs in blood. (**D**) Frequency of tumor TCRs found in blood for patients with matched tumor and blood samples by HIV/ART status (*n* = 16, pairwise Wilcoxon rank sum test). HIV^+^/ART-naive patients had higher tumor-blood overlap compared with HIV^–^ patients. Horizontal black line indicates median. (**E**) Kaplan-Meier curve of overall survival probability by tumor-blood TCR repertoire overlap (*n* = 16, Cox regression). Patients with higher tumor-blood overlap had improved survival compared with those with low overlap. Median cutoff was used to determine high versus low overlap.

To test for associations with outcome, we filtered for shared TCRs that were present in at least 5 tumor or 5 blood samples. In tumor, 2 shared TCRs were prognostic for PFS (univariate Cox regression) (Figure 4A). In blood, *n* = 17 shared TCRs were prognostic for OS and/or PFS (univariate Cox regression) (Figure 4B). In a multivariate Cox regression analysis including HIV/ART status, age, stage > 2, ECOG, and LDH, 2 blood TCRs remained significantly prognostic (CASSLDSYNEQFF: HR = 0.01, *P* = 0.004; CASSLTDTQYF: HR = 0.02, *P* = 0.005). Of those in VDJdb, prognostic shared TCRs were predicted to target CMV, EBV, HIV/HIV-1, SARS-CoV-2, dengue virus 1, and yellow fever virus (Supplemental Table 2). Multiple shared blood TCRs found in 5 or more patients may have prognostic value, even after adjusting for HIV/ART status and known prognostic covariates (29).

### Increased tumor and blood TCR repertoire overlap is positively prognostic and associates with HIV/ART status.

We next assessed prognostic value of tumor and blood TCR repertoire overlap, which may correlate with effective T cell trafficking and reinvigoration of the intratumor T cell response. There was a negative association between tumor-blood TCR repertoire overlap and age (*R* = –0.89, *P* < 0.001, Spearman correlation) (Figure 4C). HIV^+^/ART-naive tumors had 9.3-fold higher frequency of overlapping whole-blood TCRs compared with HIV^–^ tumors (*P* = 0.0079, pairwise Wilcoxon rank sum test) (Figure 4D). Patients with high overlap between tumor and whole-blood repertoires had improved OS (HR = 0.15, *P* = 0.023, Cox regression) and PFS (HR = 0.24, *P* = 0.045, Cox regression) compared with those with low overlap (Figure 4E). This trend was marginally significant after adjusting for age and HIV/ART status (OS: HR = 0.02, *P* = 0.055; PFS: HR = 0.07, *P* = 0.11; Cox regression). Increased overlap between tumor and blood TCR repertoires associated with age, HIV/ART status, and improved survival in our cohort.

## Discussion

In this study, we demonstrate the impact of HIV status and prior ART exposure on tumor TCR repertoire clonality in DLBCL. To our knowledge, this is the first description of HIV^+^ DLBCL blood and tumor TCR repertoires and the first to include both HIV^+^ and HIV^–^ patients with DLBCL from Africa. Although there is immense heterogeneity among HIV^+^ patients with DLBCL because of HIV infectious course, as well as ART regimen and response, we provide evidence that stratifying HIV^+^ patients with DLBCL by a 6-month ART duration threshold captures differences in the TCR repertoire (30). Because the TCR repertoire has predictive value for immune checkpoint inhibition (ICI) response, establishing TCR repertoire characteristics in an African cohort with considerable HIV burden is a critical step to future ICI clinical trials worldwide (14). Further, although over 7% of DLBCL deaths in the United States occur in people with HIV, these patients have historically been excluded from DLBCL molecular and biomarker studies, which limits applicability of findings from these studies (31).

In our cohort, blood TCR repertoires were enriched for TCRs with little to no clonal expansion and hyperexpanded TCRs, likely representing background immunity and clonal T cell response to infection and/or tumor, respectively. This contrasts with the tumor TCR repertoires marked by large clonal expansions, presumably from effective antitumor targeting. HIV^+^/ART-naive tumors had more clonal TCR repertoires compared with HIV^–^ and HIV^+^/ART-exp., driven by hyperexpanded clones. The increased TCR repertoire clonality in HIV^+^/ART-naive tumors is in keeping with the known impact of HIV infection on T cell expansion (19, 20). Meanwhile, prior ART restored TCR repertoire diversity in the tumor to be comparable to HIV^–^ tumors. Unlike the TCR-sequencing study of HIV^–^ DLBCL by Keane and colleagues, we did not observe a negative association between tumor clonality and outcome, even among only HIV^–^ patients (17). This may be due to sample size or differences between Australia and Malawi in a variety of nonbiological factors that impact overall outcomes and offset any impact of TCR clonality on survival. However, there may be a positive association between tumor clonality and outcome among HIV^+^ patients in our cohort. Across the whole cohort, increased TCR repertoire diversity in the blood was positively prognostic, potentially because it is a surrogate marker for overall immune health.

At the TCR level, the repertoires of HIV^+^/ART-naive tumors were more distinct from one another than from HIV^–^ or HIV^+^/ART-exp. repertoires, suggesting the lack of immune pressure in an HIV^+^/ART-naive host leads to a variety of pathways to lymphoma development and/or evolution. Alternatively, or in addition, ART may homogenize the T cell response to tumor such that it is similar to the response in HIV^–^ DLBCL. By cross-referencing the TCRs identified in our cohort with VDJdb, our study highlights known limitations of epitope species prediction, particularly in a cohort from a region that is underrepresented in sequencing studies (32). Two blood TCRs shared by 5 or more patients associated with survival after controlling for HIV/ART status, age, ECOG, stage > 2, and LDH, representing potential biomarkers. In our cohort, tumor and blood TCR repertoire overlap was associated with age and HIV/ART status. Further, there was a positive association between survival and tumor-blood TCR repertoire overlap, even after adjusting for age and HIV/ART status. Increased tumor-blood TCR repertoire clonality has been associated with improved ICI response in other malignancies (31, 33). Given the demonstrated safety and efficacy of ICI in HIV^+^ patients with non-Hodgkin lymphoma, HIV^+^/ART-naive patients may present a target population for ICIs that have so far been ineffective in HIV^–^ patients with DLBCL (34). Further, as we observe TCR reconstitution following ART, we propose that ICIs found to be effective in HIV^–^ DLBCL patients may also provide benefit for HIV^+^/ART-exp. patients.

Here, we observe differences in the tumor TCR repertoire related to systemic immunity in a DLBCL cohort. Our study is unique because of the substantial proportion of HIV^+^ DLBCL (with and without prior ART exposure) and limited number of EBV^+^ DLBCL, paired with thorough immunohistochemical data and 5-year clinical follow-up. This study is the first to our knowledge to demonstrate both HIV- and ART-related differences in the tumor TCR repertoire of DLBCL. Further work, including neoantigen prediction and studies of T cell functional status, will be necessary to elucidate potential therapeutic avenues in this key population.

## Methods

### Sex as a biological variable.

As both DLBCL and HIV affect patients regardless of sex, men and women were included in the study cohort.

### Kamuzu Central Hospital Lymphoma Study.

Based in Lilongwe, Malawi, the Kamuzu Central Hospital (KCH) Lymphoma Study is a prospective cohort that has enrolled patients with newly diagnosed lymphomas since 2013 (35). All patients received CHOP, and HIV^+^ patients continued or initiated ART. A subset of patients also received rituximab as part of a clinical trial (36). ART-experienced was defined as greater than 6 months of ART before study enrollment. For this sequencing study, all patients met the following inclusion criteria: enrolled between 2013 and 2019, at least 18 years old at enrollment, histologically confirmed de novo DLBCL, and known ART status. Patients were followed for 5 years or censored on March 20, 2023. OS was calculated from date of study enrollment to date of death or censoring, and PFS was calculated from date of study enrollment to date of progression, death, or censoring, whichever occurred first.

### Immunohistochemistry.

As previously described, diagnosis was determined by hematoxylin and eosin staining and immunohistochemistry (IHC) using antibodies as previously described: CD3 (clone PS1), CD20 (clone L26), CD30 (clone 15B3), CD45 (code NCL-L-LCA-RP), CD138 (clone MI15), BCL2 (clone bcl2/100/D5), Ki-67 (clone MM1), and TdT (clone TdT-338), all from Leica Biosystems (12, 35, 37). Additional IHC and EBER ISH were performed on a Bond platform (Leica Biosystems) according to manufacturer’s instructions. Ki-67 was quantified by light microscopy (Olympus BX43).

### DNA extraction.

We extracted DNA from 68 pretreatment formalin-fixed, paraffin-embedded (FFPE) DLBCL and 24 whole-blood samples (QIAmp DNA FFPE Advanced and Blood Mini Kits, QIAGEN) and eluted them in 30 μL and 100 μL elution buffer, respectively. After excluding samples with concentrations below 10 ng/μL by NanoDrop spectrophotometer (Thermo Fisher Scientific), *n* = 57 tumor and *n* = 21 blood samples were included in the final sequencing cohort.

### TCR sequencing.

We performed immunoSEQ Human TCRB assay according to the manufacturer’s instructions (Adaptive Biotechnologies) using 4 genomic aliquots per sample for a total input of 2 μg DNA wherever possible. Pooled libraries were quantified with the Collibri Library Quantification Kit (Invitrogen) and run on an Illumina NextSeq 500 instrument at a final concentration of 1.5 pM. Raw sequencing data were processed by Adaptive’s pipeline. Only samples that passed Adaptive’s default quality control and had more than 100 productive templates were included in the clonality analysis, and all samples were considered for remaining analyses. Only productive templates, defined as in-frame with no stop codon, were considered. Because T cell count impacts template count and varies widely based on FFPE tumor block, random downsampling was used for tumor-only and tumor-versus-blood analyses (averaged over *n* = 100 iterations). All samples were downsampled to *n* = 108 productive templates, the lowest template count above the 100 productive template threshold. Downsampling was not used for blood-only analyses, as the median template count was adequate to have minimal influence on clonality metrics.

### TCR-sequencing data analysis.

Productive Simpson clonality was calculated as the square root of Simpson’s diversity index for all productive rearrangements, ranging 0–1, with values approaching 1 indicating increased clonality. Productive maximum frequency represents the frequency of the most expanded clone in a sample, with a higher value indicating a more clonal sample. Unique productive rearrangements indicate the number of unique productive TCR rearrangements in a sample, with a higher value indicating greater diversity. The immunarch package (0.9.0) was used to generate a table of all productive TCRs (unique defined by amino acid sequence) and calculate sample overlap (intersection of 2 repertoires divided by smaller of the 2 repertoires) and relative abundance of clonal expansions by proportion of repertoire they occupy (small: 0–0.0001, medium: 0.0001–0.001, large: 0.001–0.01, or hyperexpanded: 0.01–1) (38). Tumor-blood TCR repertoire overlap was calculated by dividing the number of tumor TCRs found in blood by the total number of tumor TCRs in each sample. Epitope proportion was calculated by dividing the number of TCRs predicted to target a given species by the total number of TCRs for an individual sample. For associations between TCRs and survival, a *P* value cutoff of less than 0.05 was used to determine statistical significance. All clonotypes were investigated in VDJdb (accessed September 8, 2023), a database of annotated clonotypes from both healthy donors and patients (39).

### RNA sequencing.

RNA extraction (Omega Bio-Tek FFPE RNA extraction kit), library preparation (KAPA HyperPrep RNA with RiboErase kit), and strand-specific mRNA sequencing (Illumina NovaSeq 6000 S4) were performed by Novogene Co. For data processing, STAR 2.7.6a was used for RNA read alignment to hg38, and transcript quantification was performed using Salmon 1.4.0. Quality control was performed by creating a sorted BAM file with samtools sort 1.10 and running Picard 2.22.4 CollectRnaSeqMetrics, then running FastQC 0.11.9 on the unsorted BAM file. Counts were normalized using upper quartile normalization and log_2_ transformation. We categorically assigned cell of origin as GC, ABC, and unclassified, according to the algorithm by Wright et al. (7, 40). We also generated a continuous value for cell of origin as previously described (12). HLA types were predicted from the sorted BAM files using arcasHLA (v0.5.0, default parameters) (41).

### Statistics.

To test associations between bulk clonality and clinical/demographic variables, we used Kruskal-Wallis test, pairwise Wilcoxon rank sum test, or Spearman correlation coefficient. BH correction was used for multiple comparisons, as indicated in the text by p.adj. Fold-change refers to median fold-change unless otherwise specified. For survival analysis, binary variables were generated based on median cutoff. HRs were estimated by Cox regression. Analyses were performed in R 4.2.0 using dplyr, tidyverse, and survival packages.

### Study approval.

The KCH Lymphoma Study (NCT02835911) was approved by the University of North Carolina Institutional Review Board and Malawi National Health Sciences Research Committee, Lilongwe, Malawi. All patients provided written informed consent before enrollment.

### Data availability.

Processed TCR-sequencing data will be available on the immuneAccess database upon publication. RNA-sequencing counts are available on NCBI GEO (GSE262621). Values included in all figures are available in the Supporting Data Values XLS file.

## Author contributions

SMR, AMX, AM, SG, and YF conceived and designed the analyses; SMR performed TCR sequencing and analyzed the data; SMR, JC, and AMX interpreted the data; SMR drafted the manuscript; JC and YF revised the manuscript; MP, EK, and KP acquired clinical data; TT, M Mponda, M Mulenga, and YF acquired pathologic data; AB, JG, HG, MC, BD, MP, AK, and SG provided intellectual contributions throughout data collection and analysis; and all authors provided edits and final approval of the manuscript.

## Supplementary Material

Supplemental data

Supporting data values

## Figures and Tables

**Table 1:**
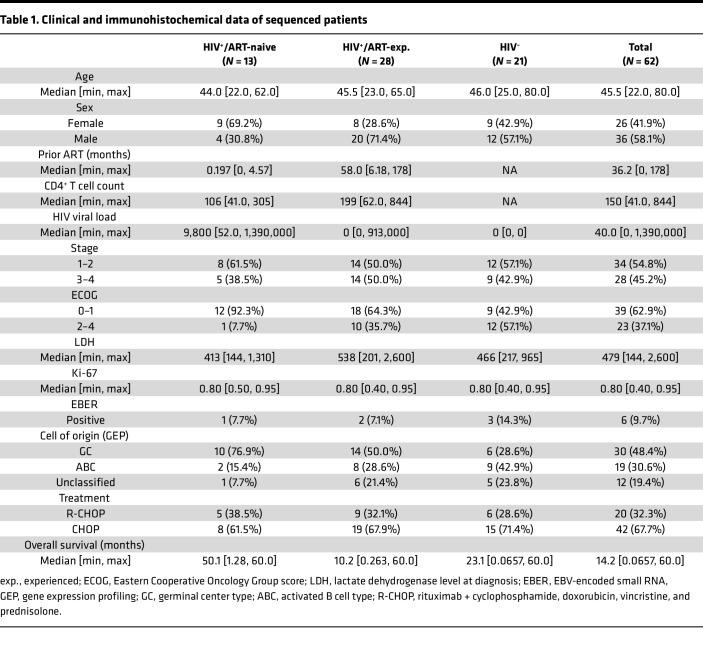
Clinical and immunohistochemical data of sequenced patients

**Table 2:**
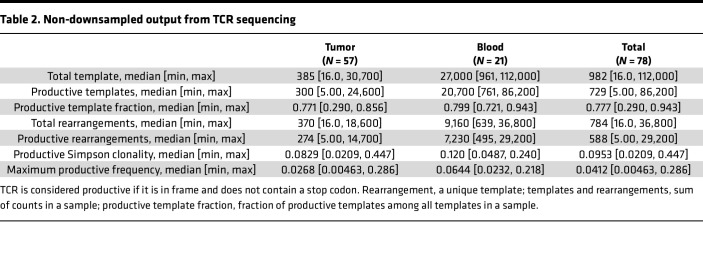
Non-downsampled output from TCR sequencing

## Version 1 05/23/2024

In-Press Preview

## Version 2 07/08/2024

Electronic publication
