# Supplementary material for: HIV infection and ART exposure affect tumor TCR repertoire of diffuse large B cell lymphoma
Source: JCI Insight. 2024 May 23;9(13):e180771. doi: 10.1172/jci.insight.180771 (PMC11383373; doi:10.1172/jci.insight.180771)
Supplement: Supplemental data [file jciinsight-9-180771-s139.pdf]

HIV infection and ART exposure impact tumor T-cell receptor repertoire of diffuse large B-cell lymphoma

Sophia M. Roush[1], Jenny Coelho[1], Alexander M. Xu[2], Kaushik Puranam[3], Marriam Mponda[3,4], Edwards Kasonkanji[3,4], Maurice Mulenga[3,4], Tamiwe Tomoka[3,4], Jonathan Galeotti[1,5], Amy Brownlee[1], Hormas Ghadially[6], Maganizo Chagomerana[3,4], Blossom Damania[5,7], Matthew Painschab[3,4,5,8], Akil Merchant[2,9], Satish Gopal[10], Yuri Fedoriw[1,3,4,5]

[1]Department of Pathology and Laboratory Medicine, School of Medicine, University of North Carolina, Chapel Hill, NC, USA, [2]Samuel Oschin Comprehensive Cancer Institute, Cedars-Sinai Medical Center, Los Angeles, CA, USA, [3]University of North Carolina Project Malawi, Lilongwe, Malawi, [4]University of Malawi College of Medicine, Lilongwe, Malawi, [5]University of North Carolina Lineberger Comprehensive Cancer Center, Chapel Hill, NC, USA, [6]Department of Pathology, School of Medicine and Oral Health, Kamuzu University of Health Sciences, Lilongwe, Malawi, [7]Department of Microbiology and Immunology, School of Medicine, University of North Carolina, Chapel Hill, North Carolina, USA, [8]Division of Hematology, School of Medicine, University of North Carolina, Chapel Hill, NC, [9]Division of Hematology and Oncology, Department of Medicine, Cedars-Sinai Medical Center, Los Angeles, CA, USA, [10]National Cancer Institute Center for Global Health, Rockville, MD, USA

Conflict-of-Interest:

The authors have declared that no conflict of interest exists.

Supplemental Table 1.

	Tumor		Blood	
	Both Analyses (N=35)	TCR Specificity Only (N=22)	Both Analyses (N=17)	TCR Specificity Only (N=4)
**Age**				
Median [Min, Max]	44.0 [22.0, 80.0]	45.5 [25.0, 62.0]	43.0 [22.0, 79.0]	53.0 [23.0, 57.0]
**Sex**				
Female	12 (34.3%)	12 (54.5%)	10 (58.8%)	1 (25.0%)
Male	23 (65.7%)	10 (45.5%)	7 (41.2%)	3 (75.0%)
**HIV/ART Status**				
HIV+/ART-naive	8 (22.9%)	3 (13.6%)	7 (41.2%)	0 (0%)
HIV+/ART-exp.	15 (42.9%)	12 (54.5%)	4 (23.5%)	3 (75.0%)
HIV-	12 (34.3%)	7 (31.8%)	6 (35.3%)	1 (25.0%)
**Prior ART (months)**				
Median [Min, Max]	41.4 [0, 178]	26.1 [0, 111]	2.38 [0, 176]	119 [78.6, 178]
**CD4 Count**				
Median [Min, Max]	199 [41.0, 844]	145 [42.0, 385]	189 [42.0, 387]	249 [68.0, 385]
**HIV Viral Load**				
Median [Min, Max]	59.0 [0, 167000]	0 [0, 1390000]	52.0 [0, 1390000]	0 [0, 37300]
**Stage**				
1-2	18 (51.4%)	15 (68.2%)	7 (41.2%)	3 (75.0%)
3-4	17 (48.6%)	7 (31.8%)	10 (58.8%)	1 (25.0%)
**ECOG**				
0-1	21 (60.0%)	15 (68.2%)	11 (64.7%)	2 (50.0%)
2-4	14 (40.0%)	7 (31.8%)	6 (35.3%)	2 (50.0%)
**LDH**				
Median [Min, Max]	478 [144, 2510]	436 [220, 2600]	433 [144, 1310]	996 [320, 2600]
**Ki67**				
Median [Min, Max]	0.80 [0.50, 0.95]	0.80 [0.40, 0.95]	0.80 [0.50, 0.95]	0.90 [0.90, 0.95]
**EBER**				
Positive	2 (5.7%)	2 (9.1%)	3 (17.6%)	1 (25.0%)
**Cell-of-Origin (GEP)**				
GC	17 (48.6%)	11 (50.0%)	9 (52.9%)	3 (75.0%)
ABC	12 (34.3%)	5 (22.7%)	4 (23.5%)	1 (25.0%)
Unclassified	5 (14.3%)	6 (27.3%)	3 (17.6%)	0 (0%)
**Treatment**				
R-CHOP	0 (0%)	0 (0%)	0 (0%)	0 (0%)
CHOP	0 (0%)	0 (0%)	0 (0%)	0 (0%)
**Overall Survival (months)**				
Median [Min, Max]	19.4 [0.0657, 60.0]	28.8 [1.28, 60.0]	12.6 [1.28, 60.0]	14.9 [7.92, 60.0]

Clinical and immunohistochemical data of sequenced patients stratified by tissue type and analysis inclusion. ECOG=Eastern Cooperative Oncology Group score; LDH= lactate dehydrogenase level at diagnosis; GEP=gene expression profiling, ABC= activated B-cell type, GC= germinal-center type

Supplemental Table 2. Prognostic shared TCRs.

	Overall Survival			Progression-Free Survival			Species
	P	HR	95% CI	P	HR	95% CI	
**Blood**							
CASSLAGGYEQYF	0.07	0.24	0.05-1.11	**0.03**	0.19	0.04-0.86	CMV, SARS-CoV-2
CASSLAGGYNEQFF	0.07	0.30	0.08-1.11	**0.03**	0.23	0.06-0.84	NA
CASSLDSYNEQFF	**0.04**	0.20	0.04-0.92	**0.02**	0.16	0.03-0.71	CMV
CASSLGAGYEQYF	**0.03**	0.19	0.04-0.89	0.07	0.31	0.09-1.11	NA
CASSLGGGYTF	**0.04**	0.11	0.01-0.86	0.05	0.23	0.05-1.01	HIV-1
CASSLGGNTEAFF	0.05	0.32	0.10-0.98	0.07	0.38	0.13-1.10	SARS-CoV-2
CASSLGNYGYTF	0.08	0.16	0.02-1.22	0.05	0.13	0.02-0.97	NA
CASSLGRGYEQYF	**0.04**	0.12	0.02-0.91	0.08	0.25	0.06-1.15	EBV
CASSLGSYEQYF	0.08	0.32	0.09-1.16	**0.04**	0.25	0.07-0.92	CMV, DENV1
CASSLGTDTQYF	**0.01**	0.18	0.05-0.61	**0.01**	0.23	0.07-0.70	CMV
CASSLGTNTEAFF	**0.02**	4.44	1.25-15.74	**0.01**	5.44	1.42-20.83	NA
CASSLSYEQYF	**0.03**	0.19	0.04-0.89	0.07	0.31	0.09-1.11	HIV-1
CASSLTDTQYF	**0.04**	0.29	0.09-0.95	0.06	0.36	0.12-1.06	CMV, EBV
CASSPQGYEQYF	**0.04**	0.12	0.02-0.91	0.08	0.25	0.06-1.15	YFV
CASSPSTDTQYF	0.07	0.24	0.05-1.11	**0.03**	0.19	0.04-0.86	NA
CASSPSYEQYF	**0.03**	0.19	0.04-0.89	**0.02**	0.15	0.03-0.69	EBV
CASSPTGNTEAFF	**0.02**	0.17	0.04-0.77	0.05	0.28	0.08-1.01	CMV
**Tumor**							
CASSSSQGAGNTIYF	0.08	2.58	0.90-7.42	**0.02**	3.13	1.19-8.23	NA
CSVDAGDAGNTIYF	0.06	2.70	0.94-7.75	**0.02**	3.27	1.25-8.58	NA

Associations of shared TCRs with survival and prognostic covariates. Univariate Cox regression was used to calculate p-value, hazard ratio (HR) and 95% confidence interval (95% CI) for associations with overall and progression-free survival. p-values <0.05 are bolded. Species refers to epitope the TCR is predicted to bind per VDJdb; CMV=Cytomegalovirus, HIV-1=Human Immunodeficiency Virus 1, YFV= Yellow Fever Virus, EBV=Epstein-Barr virus, DENV1= Dengue Virus 1, SARS-CoV-2= Severe acute respiratory syndrome coronavirus 2, NA=TCR not identified in VDJdb

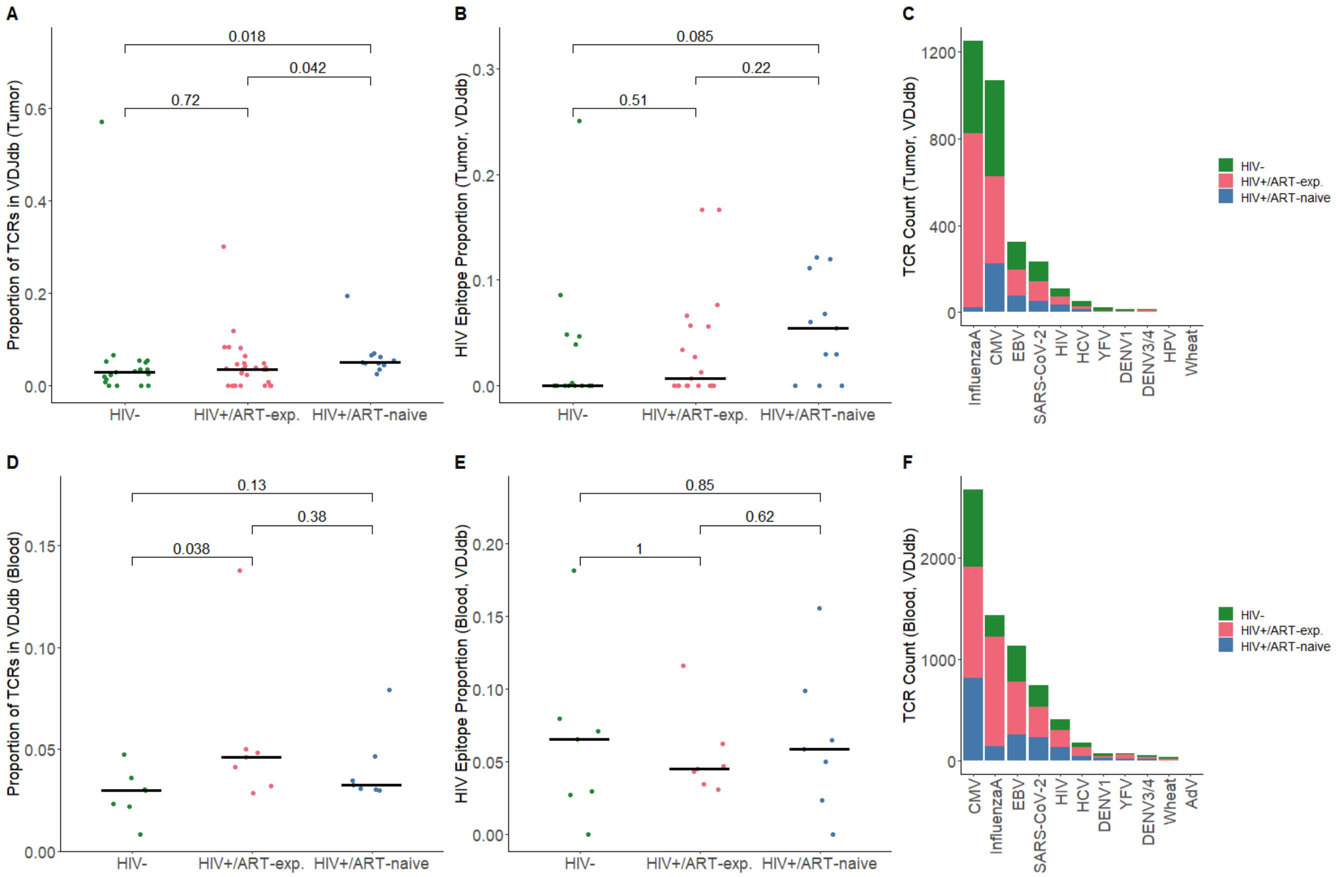

Supplemental Figure 1. TCRs from our cohort are underrepresented in VDJdb database. A) Proportion of tumor TCRs in our samples identified by VDJdb database (n=57, pairwise Wilcoxon rank sum test). **B)** Proportion of tumor TCRs predicted to target HIV by HIV/ART status (n=57, pairwise Wilcoxon rank sum test). **C)** Most common predicted targets of tumor TCRs found in VDJdb. **D)** Proportion of blood TCRs in our samples identified by VDJdb database (n=21, pairwise Wilcoxon rank sum test). **E)** Proportion of blood TCRs predicted to target HIV by HIV/ART status (n=21, pairwise Wilcoxon rank sum test). **F)** Most common predicted targets of blood TCRs found in VDJdb. Horizontal black line indicates median.

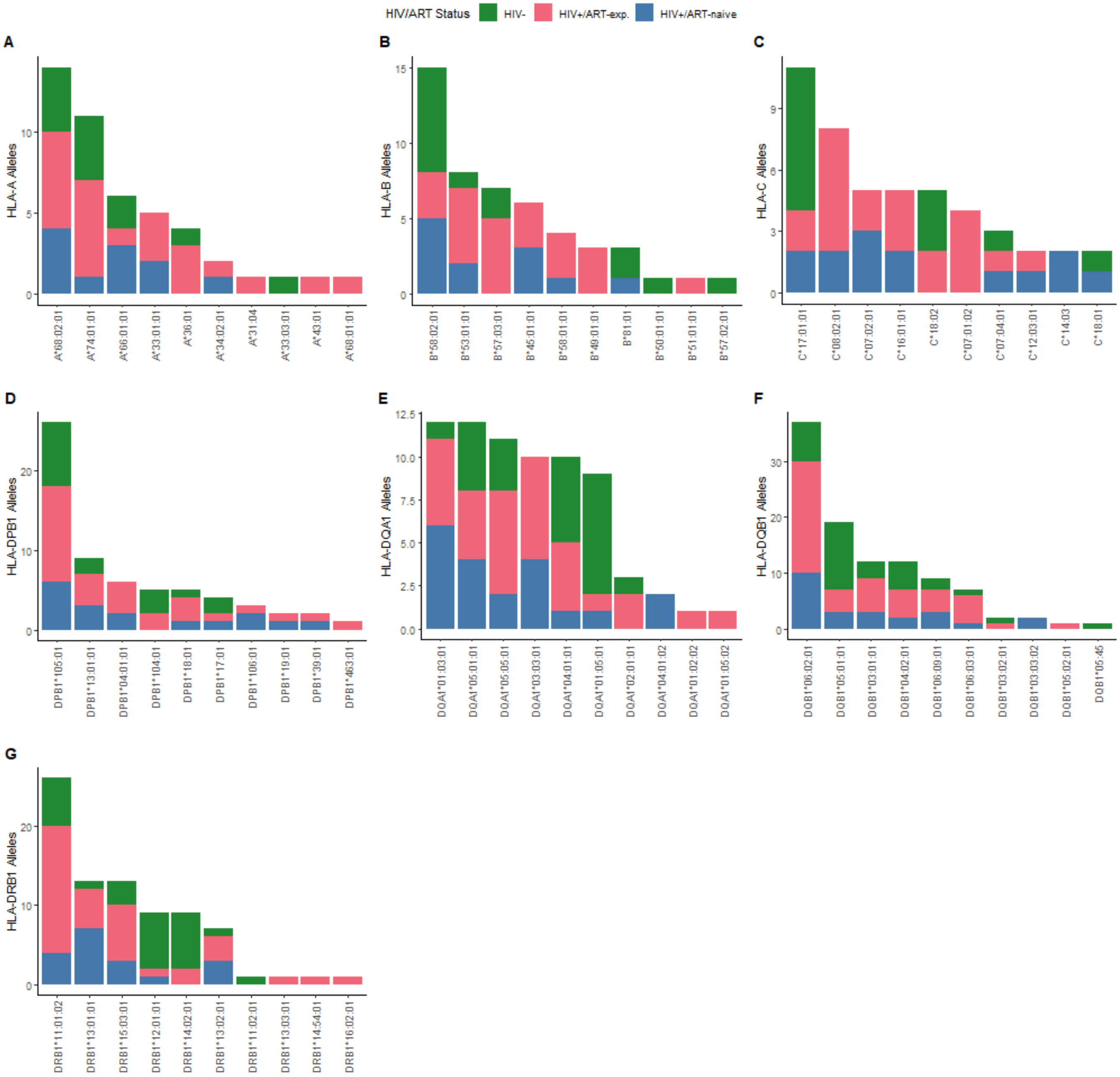

Supplemental Figure 2. HLA types by HIV/ART status. Distribution of **A)** HLA-A, **B)** HLA-B, **C)** HLA-C, **D)** HLA-DPB1, **E)** HLA-DQA1, **F)** HLA-DQB1 and **G)** HLA-DRB1 alleles by HIV/ART status.
